# Visualization of the renal vein during pyelography after nephrostomy: a case report

**DOI:** 10.1186/1752-1947-4-93

**Published:** 2010-03-23

**Authors:** Abdallah Geara, Leila Kamal, Badiaa El-Imad, Suzanne El-Sayegh

**Affiliations:** 1 Department of Internal Medicine, Staten Island University Hospital, Seaview Avenue, Staten Island, New York, USA

## Abstract

**Introduction:**

We present a case of pyelovenous backflow after nephrostomy. To the best of our knowledge, this is the first documented case of renal vein visualization after a nephrostomic placement.

**Case presentation:**

A 55-year-old Caucasian man presented with symptoms of pyelonephritis with an obstructing ureteral stone. A nephrostomy was performed. During an injection of contrast agent in his left caliceal system, his left renal vein was visualized. A repeat pyelography with an injection contrast material at low pressure failed to show the same finding. This radiological finding is due to the occurrence of "pyelovenous backflow".

**Conclusion:**

This phenomenon is usually described in the setting of renal vein thrombosis, renal vein hypertension due to the "nutcracker phenomenon", or a reduced renal blood flow. Examination by microscopy shows the presence of tears in the fornix of the pelvic cavity that extend into the kidney parenchyma. Five types of renal backflow are described in the literature: pyelovenous, pyelolymphatic, pyelotubular, pyelointerstitia and pyelosinus. Injection of contrast material at high pressure may cause a fornix to flow into the tubules, or cause its rupture and flow into the venous system.

## Introduction

We present a case of interventional radiology that showed a very interesting finding during nephrostomy. The images during the procedure were very alarming for the radiologist who requested a critical care evaluation. The initial finding was in favor of an iatrogenic complication.

## Case presentation

A 55-year-old Caucasian man presented with a three-day fever, chills and abdominal pain. His medical history indicated that he had hypertriglyceridemia and hypertension. His physical examination was positive for fever (38.3°C), tachycardia (110 beats/minute) and tenderness upon palpation of his left flank. His initial laboratory evaluation showed leukocytosis (18,600), acute renal failure (creatinine 3.8 mg/dL; baseline creatinine 1.2 mg/dL) and numerous white blood cells (WBCs) in his urine.

A computed tomography (CT) scan of his abdomen and pelvis showed the presence of left hydronephrosis and an obstructing ureteral stone with a diameter of 1.5 cm. Our patient was diagnosed with left pyelonephritis. He was immediately commenced on broad-spectrum antibiotics. A left percutaneous nephrostomy was also immediately performed on our patient. His sepsis and acute renal failure subsequently improved.

**Figure 1:**
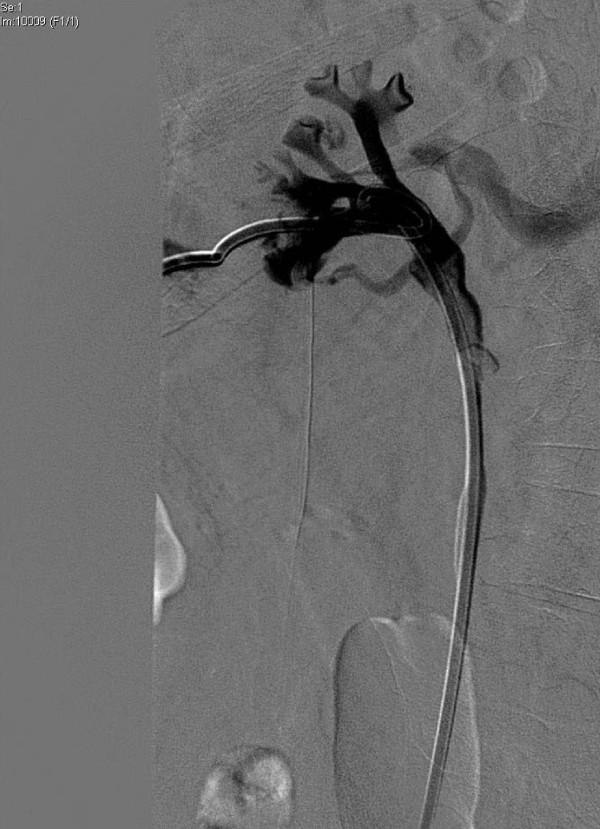
**Pyelovenous backflow**. During pyelography, the renal vein was visualized after the injection of contrast material at high pressure through the nephrostomy.

After a ureteral stent placement, our patient underwent an internalization of the nephrostomy. During an injection of contrast agent in his left caliceal system, we were able to visualize his left renal vein (Figure 1). At this point, however, our patient was clinically stable, had no hematuria, and maintained a stable hemoglobin level. Finding no convenient explanation for this interesting radiological finding, we initially suspected an iatrogenic renal veno-caliceal fistula.

## Discussion

Veno-caliceal fistulas are rarely discussed in the literature. They usually occur in specific circumstances, such as when a venous and caliceal perforation occurs simultaneously, thus leading to a communication of the two systems. Patients usually present with gross hematuria during trauma. One case report described a veno-caliceal fistula in a patient with intermittent gross hematuria and with no known history of trauma [1]. Veno-caliceal fistula can also occur as a urological complication due to graft ureteric stricture after kidney transplantation. One case report presented a kidney transplant patient with pseudorenal failure and graft ureteral stricture. Since the intra-caliceal pressure was stronger than the venous pressure as a result of the ureteral stricture, the urine recirculated into our patient's blood. This in turn increased the serum creatinine level of our patient without causing the alteration of his kidney functions [2].

Another explanation for this radiological finding is a "pyelovenous backflow". This phenomenon is described in the setting of renal vein thrombosis, renal vein hypertension due to the "nutcracker phenomenon" or reduced renal blood flow [3]. This condition is seen on microscopy as tears in the fornix of the pelvic cavity that extend into the kidney parenchyma. In rabbits with unilateral renal vein occlusion, capsular, perihilar, periureteral, and retroperitoneal collateral vein networks and lymphatic channels on the venous occluded side can be visualized by pyelography [3].

The literature describes five types of renal backflow: pyelovenous, pyelolymphatic, pyelotubular, pyelointerstitial and pyelosinus [4]. The presence of chronic hydronephrosis contributes to tears in the caliceal fornix, which usually occur in an ischemic kidney [5]. Contrast material injected at high pressure may flow into the tubules or may rupture a fornix and flow into the venous system.

## Conclusion

Since our patient did not have any episode of gross hematuria following the internalization of nephrostomy, the possibility of his having a veno-caliceal fistula was minimal. The repeat pyelography, which allowed for the injection of contrast material at low pressure, failed to visualize his renal vein. CT scan of his abdomen and pelvis with intravenous contrast did not show any renal vein thrombosis.

To the best of our knowledge, this report describes the first reported case of pyelovenous backflow that was visualized after a nephrostomy.

## Consent

Written informed consent was obtained from our patient for publication of this case report and any accompanying images. A copy of the written consent is available for review by the Editor-in-Chief of this journal.

## Competing interests

The authors declare that they have no competing interests.

## Authors' contributions

AG and LK analyzed and interpreted data from our patient's imaging findings and medical care. BEI and SES were major contributors in reviewing the literature and in writing the manuscript. All authors read and approved the final manuscript.
